# A case of Klippel–Feil syndrome with adult-onset neurological symptoms

**DOI:** 10.1016/j.radcr.2026.06.132

**Published:** 2026-07-18

**Authors:** Karthik Raja Ravichandran, James G. Wilcox, Christopher L. Newman

**Affiliations:** a Indiana University School of Medicine, 340 West 10th Street Fairbanks Hall, Suite 6200 Indianapolis, IN 46202, USA; b Department of Radiology and Imaging Sciences, Indiana University, 702 North Barnhill Drive, Room 1053, Indianapolis, IN, 46202, USA; c Department of Family Medicine, Indiana University School of Medicine, 980 Indiana Avenue Lockefield Village 1164 Indianapolis, IN, 46202, USA; d Family Medicine, United Arab Emirates University, Al Ain, United Arab Emirates

**Keywords:** Klippel–Feil syndrome, Hemivertebra, Cervical radiculopathy, Thoracic outlet syndrome

## Abstract

Klippel–Feil syndrome is a rare congenital anomaly characterized by fusion of cervical vertebrae. We describe the case of a 45-year-old female presenting with nonspecific neurological symptoms which was discovered to be due to undiagnosed Klippel–Feil syndrome. This case is unique due to presentation in adulthood as this condition is usually diagnosed in childhood due to visual asymmetries. Additionally, we highlight the value of multimodal imaging and diagnostics when attempting to delineate overlapping pathologies related to the cervical spine.

## Introduction

Klippel–Feil syndrome (KFS) is a rare congenital disorder characterized by failure of normal segmentation of the cervical somites, resulting in nonsegmented or congenitally fused cervical vertebrae [1,2]. The syndrome was first described by Maurice Klippel and André Feil in 1912 in a patient with the classic clinical triad of a short neck, low posterior hairline, and restricted cervical range of motion [3]. However, the complete triad is present in only a subset of patients, and clinical presentation varies considerably depending on the number, level, and pattern of involved vertebral segments [1,4].

The condition is believed to arise during early embryologic development, particularly between the third and eighth weeks of gestation, and has been associated with abnormal somitic segmentation, vascular disruption, aberrant neural tube development, and genetic factor [1,5]. The most commonly involved cervical levels are C2-C3 and C5-C6 [1,4]. Although the musculoskeletal system is most predominantly affected, KFS may also be associated with abnormalities involving the cardiovascular, urogenital, respiratory, auditory, and neurologic systems [1,4,6]. Therefore, imaging is important not only for defining the vertebral segmentation anomalies but also for evaluating the spinal canal, neural foramina, vascular structures, and associated extraspinal abnormalities [4,6].

Radiographs may identify congenital fusion, block vertebrae, hemivertebrae, scoliosis, or secondary degenerative changes, while computed tomography (CT) and computed tomography angiography (CTA) provide detailed assessment of osseous anatomy and vascular structures [4,6]. Magnetic resonance imaging (MRI) is particularly useful for evaluating the spinal cord, nerve roots, intervertebral discs, neural foramina, and surrounding soft tissues, as well as identifying associated abnormalities such as syringomyelia, spinal stenosis, or nerve root compression [4,6]. Ultrasound can be helpful for identifying associated abdominal anomalies, such as horseshoe kidney or uterine duplication. This case highlights the value of multimodal imaging in an adult patient with previously undiagnosed KFS and overlapping neurologic and musculoskeletal symptoms.

## Case presentation

We present the case of a 45-year-old female with a past medical history of asthma and lactose intolerance who presented with bilateral hand numbness, migraines, neck pain, and left upper extremity pain. The patient described the neck pain as a sharp pain radiating down the left arm into the hand. The left upper extremity pain was positional and got worse at night. This started gradually but had been going on for about 5 months. On physical exam, the patient had upright posture with no visual deformity on inspection. There was tenderness on the cervical spine with limited flexion and extension of the neck. A positive Spurling test was observed on the left side. A positive Tinel sign was noted on median nerve compression testing bilaterally. The patient had a positive Adson and Roos test concerning for thoracic outlet syndrome as well as a positive Cozen’s test on the left elbow signifying lateral epicondylitis. Radiographic imaging of the cervical spine demonstrated fusion anomalies and hemivertebrae in the lower cervical and upper thoracic spine (consistent with a type II Klippel–Feil deformity) with mild superimposed degenerative changes (Fig. 1).

**Fig. 1:**
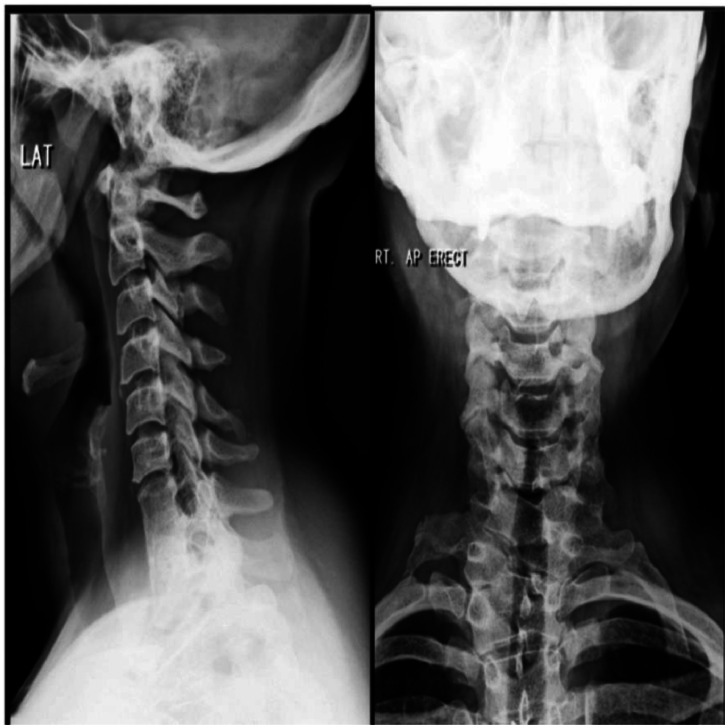
Anteroposterior and lateral radiographic images of the cervical spine demonstrate congenital segmentation anomalies at the cervicothoracic junction, including hemivertebrae and vertebral body fusion anomalies consistent with Klippel–Feil syndrome. Mild multilevel spondylodegenerative changes are also present, including disc space narrowing and osteophyte formation.

MRI of the cervical spine was obtained to better characterize the neurovascular structures associated with the fusion anomalies and to identify a focal source for the thoracic outlet syndrome symptoms. MRI clarified the anatomical relationships of the fusion anomalies and hemivertebrae. Specifically, there was a left C7 hemivertebra and a right T1 hemivertebra with an oblique vertebra extending from C7 on the right to T1 on the left (Fig. 2). Additionally, the images demonstrated an atypical relationship of the posterior arches. Specifically, the pedicle of the left C7 hemivertebra is connected to the right C7 portion of the oblique vertebra, and the pedicle of the right T1 hemivertebra is connected to the left T1 portion of the oblique vertebra. There were mild superimposed spondylodegenerative changes with disc protrusions at C3-C4 and C6-C7.

**Fig. 2:**
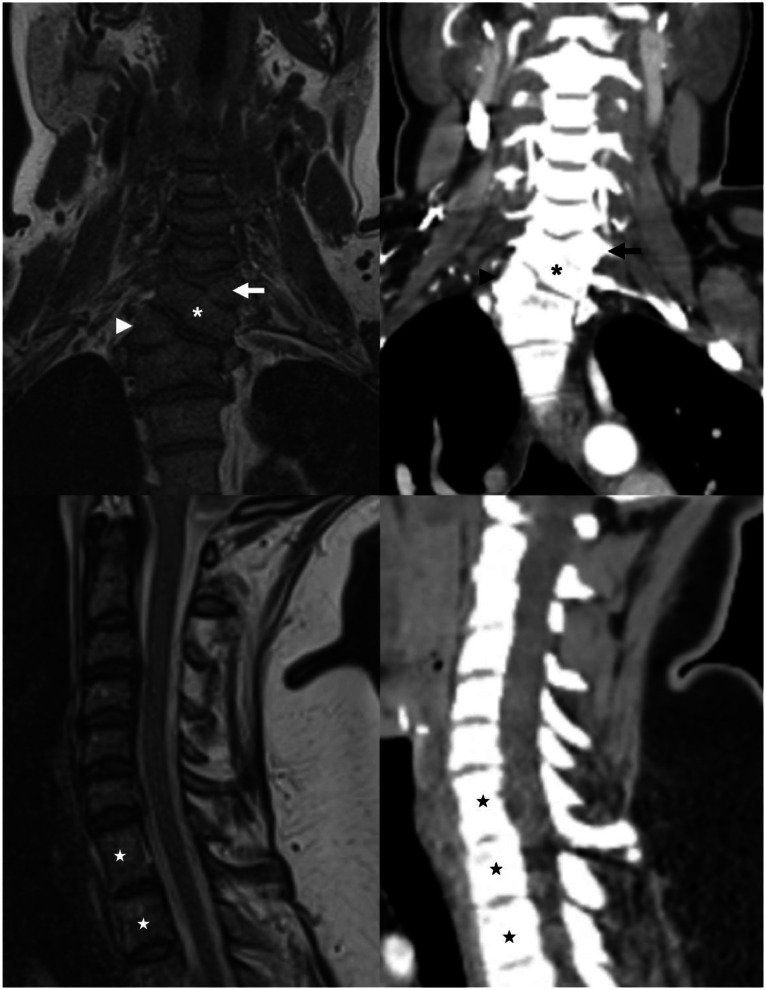
Coronal T2 MRI (top left) and CTA (top right) images of the cervical spine demonstrate complex congenital vertebral anomalies at the cervicothoracic junction, including a left C7 hemivertebra (solid arrows), right T1 hemivertebra (arrowheads), and an oblique vertebral segment extending from C7 on the right to T1 on the left (asterisks). Sagittal T2 MRI (bottom left) and CTA (bottom images) demonstrate elongated lower cervical and upper thoracic block vertebrae (stars) resulting from the fusion anomalies.

Additionally, there was a pseudoarthrosis between the left T1 and T2 ribs with associated compression of the left T1 nerve root. CTA imaging was performed to evaluate for vascular compression. The fusion anomalies and hemivertebrae were similar to prior imaging, but there was no evidence of vascular compression (Fig. 3). This pseudoarthrosis provides a plausible structural correlate for the patient’s left upper extremity symptoms.

**Fig. 3:**
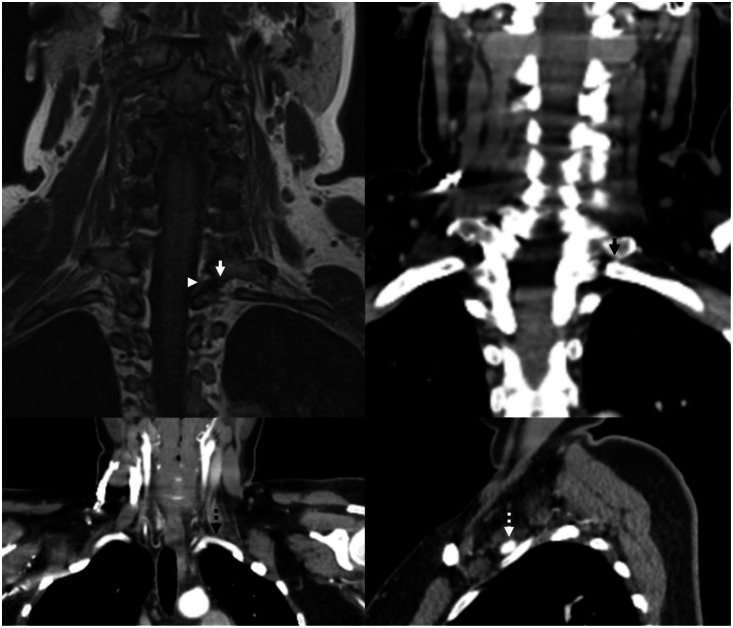
Coronal T1 MRI (top left) and CTA (top right) images demonstrate a pseudoarthrosis between the first and second left ribs (solid arrows) with associated narrowing and compression of the adjacent left T1 nerve root (arrowhead). This finding provides a plausible structural correlate for the patient’s left upper extremity symptoms. Coronal (bottom left) and sagittal (bottom right) CTA images demonstrate a patent left subclavian artery (dashed arrows).

Radiographs identified the initial cervicothoracic segmentation anomalies and prompted cross-sectional evaluation. MRI better characterized the complex vertebral anatomy, posterior arch relationships, disc protrusions, and relationship of the congenital anomalies to adjacent nerve roots. CTA confirmed the osseous configuration and demonstrated patency of the left subclavian artery without fixed vascular compression. Together, these modalities helped distinguish structural nerve root irritation from arterial thoracic outlet syndrome.

Overall, the imaging demonstrated a complex cervicothoracic congenital segmentation anomaly with left C7 and right T1 hemivertebrae, an oblique vertebral segment extending from C7 to T1, elongated lower cervical and upper thoracic block vertebrae, and pseudoarthrosis between the first and second left ribs. MRI demonstrated narrowing and compression of the adjacent left T1 nerve root, while CTA confirmed patency of the left subclavian artery without fixed arterial compression.

Symptomatic management involved bilateral wrist splints, physical therapy, and nonsteroidal anti-inflammatory drug gel 2-3 times daily. For her migraines, she was prescribed Rimegepant. Botox and epidural injections were discussed as options for her cervical radiculopathy. A nerve conduction study was performed, which demonstrated evidence of right carpal tunnel syndrome but normal left median nerve conduction which was unexpected given the left hand symptoms (Fig. 4). Later, electromyography demonstrated left C4 radiculopathy, which may have been related to the C3-C4 disc protrusion. However, the C6 and C7 conduction studies were normal.

**Fig. 4:**
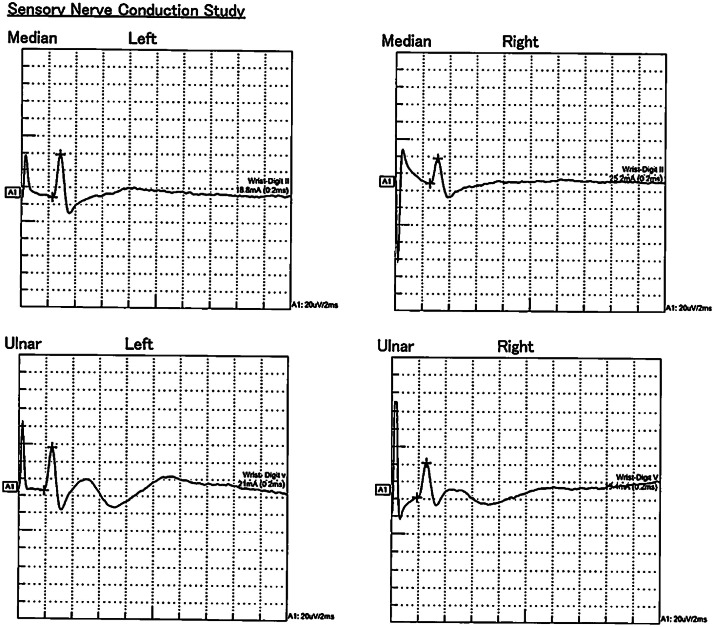
Nerve conduction study showing right axonal median neuropathy and normal ulnar sensation.

## Discussion

### Imaging findings by modality

This case demonstrates the complementary role of radiographs, MRI, and CTA in evaluating an adult presentation of KFS [1,2]. Initial cervical spine radiographs demonstrated congenital segmentation anomalies at the cervicothoracic junction with hemivertebrae, vertebral fusion anomalies, and mild multilevel spondylodegenerative change. These findings prompted further cross-sectional evaluation because the radiographs demonstrated more than isolated degenerative osteophyte formation.

MRI provided further characterization of the complex congenital anatomy. The study demonstrated a left C7 hemivertebra, right T1 hemivertebra, and an oblique vertebral segment extending from C7 on the right to T1 on the left. MRI also demonstrated abnormal posterior arch and pedicle relationships, mild superimposed degenerative disc protrusions at C3-C4 and C6-C7, and pseudoarthrosis between the first and second left ribs with narrowing and compression of the adjacent left T1 nerve root. These findings provided a plausible structural explanation for the patient’s left upper extremity symptoms.

CTA was performed because physical examination raised concern for thoracic outlet syndrome. CTA confirmed the congenital osseous abnormalities but showed no fixed vascular compression or arterial abnormality. The left subclavian artery remained patent. Therefore, the positive Adson sign was interpreted as a nonspecific provocative finding rather than diagnostic evidence of arterial thoracic outlet syndrome [7,8].

## Classification

Multiple classification systems have been proposed for KFS, including the original KFS, Clarke, and the Samartzis classifications [[9], [10]]. These systems help describe the extent and pattern of congenital vertebral nonsegmentation and may provide prognostic information regarding the risk of axial symptoms, radiculopathy, or myelopathy. The Clarke classification is a historical classification, which characterized genotypic and phenotypic heterogeneity, but this has been largely replaced by the KFS and Samartzis classifications. The Samartzis classification system carries prognostic value and describes the anatomical pattern of vertebral fusion but does not inherently determine the genotype as with the original KFS classification (Table 1) [11]. In the present case, the cervicothoracic segmentation anomalies and associated radicular symptoms are most relevant when interpreted alongside the imaging findings rather than as an isolated classification label.

**Table 1:** KFS classification.

	Original KFS	Samartzis
Type I	Continuous fusion of cervical and potentially upper thoracic vertebrae. This is associated with a sporadic mutation	Single congenital fusion of cervical segment with symptoms affecting the axial spine
Type II	Fusion of 2-3 cervical vertebrae. This is associated with an autosomal dominant mutation in the GDF3 or GDF6 gene	Multiple, noncontiguous congenitally fused segments which lead to peripheral myelopathy or radiculopathy
Type III	Cervical fusion in addition to fusion in parts of the thoracic or lumbar spine. This is associated with an autosomal recessive mutation in the MEOX1 gene	Multiple, contiguous congenitally fused segments which lead to peripheral myelopathy or radiculopathy

The table was independently created by the authors from cited references.

## Differential diagnoses and clinicoradiological correlation

The patient’s symptoms were polysymptomatic and not all were attributed directly to KFS. Neck pain, restricted cervical motion, radicular symptoms, cervicothoracic segmentation anomalies, and left T1 nerve root compression were considered most directly related to the congenital cervicothoracic abnormality. In contrast, migraines, right median neuropathy, and left lateral epicondylitis were interpreted as overlapping or separate diagnoses. The normal CTA and absence of secondary vascular signs, such as edema, discoloration, ischemic change, or intrinsic hand muscle atrophy, made arterial and venous thoracic outlet syndrome less likely [7,8]. The electrodiagnostic findings also supported overlapping pathology, including right carpal tunnel syndrome and left C4 radiculopathy.

KFS may mimic or coexist with other musculoskeletal and neurologic disorders including degenerative cervical radiculopathy, thoracic outlet syndrome, peripheral entrapment neuropathies such as carpal tunnel syndrome or ulnar neuropathy, and mechanical axial neck pain, making clinicoradiological correlation important [1,12,13]. In the present case, multimodal imaging helped distinguish congenital segmentation anomalies and nerve root irritation from peripheral entrapment neuropathy, degenerative cervical radiculopathy, and vascular thoracic outlet syndrome.

## Comparison with previously published cases

Thoracic outlet syndrome has rarely been reported in association with KFS [14,15]. Previously published cases have described thoracic outlet symptoms in the setting of congenital skeletal abnormalities, including cervical ribs or anomalous cervicothoracic anatomy [14,15]. In the present case, clinical examination raised concern for thoracic outlet syndrome, but CTA did not demonstrate arterial compression. Instead, MRI showed pseudoarthrosis between the first and second left ribs with adjacent left T1 nerve root compression. This supports a predominantly neurogenic and structural mechanism rather than arterial thoracic outlet syndrome.

## Treatment options

Management of KFS depends on symptom severity, neurological involvement, instability, and associated anomalies [1,12,13]. Nonoperative treatment may include activity modification, physical therapy, analgesia, nonsteroidal anti-inflammatory medications, splinting for peripheral entrapment neuropathies, and clinical monitoring [1,12,13]. Surgical intervention may be considered for progressive neurological deficit, refractory pain, significant stenosis, instability, or deformity [1,12,13]. In this patient, treatment was conservative and symptom-directed, including bilateral wrist splints, physical therapy, topical nonsteroidal anti-inflammatory drug therapy, and discussion of injection-based options for cervical radicular pain.

## Limitations

This case has several limitations. First, it represents a single adult presentation of KFS, limiting generalizability. Second, the posterior hairline was not documented, so only restricted cervical range of motion was clearly documented from the classic clinical triad. Third, although CTA excluded fixed vascular compression, the lack of imaging with abduction limits evaluation for positional vascular compromise [7,8]. Finally, the patient had overlapping conditions, including right carpal tunnel syndrome, cervical radiculopathy, migraines, and lateral epicondylitis, which limited the ability to attribute all symptoms directly to KFS.

## Conclusion

KFS may present in adulthood with overlapping neurological and musculoskeletal symptoms. This case highlights the value of multimodal imaging in characterizing complex cervicothoracic segmentation anomalies, identifying potential nerve root involvement, and excluding fixed vascular compression. Careful clinicoradiological correlation is essential to distinguish symptoms related to congenital anatomy from coexisting conditions.

## Patient consent

Written informed consent was obtained from the patient for publication of this case report.

## References

[1] Samartzis D, Kalluri P, Herman J, Lubicky JP, Shen FH (2016). “Clinical triad” findings in pediatric Klippel-Feil patients. Scoliosis Spinal Disord.

[2] Tracy MR, Dormans JP, Kusumi K (2004). Klippel-Feil syndrome: clinical features and current understanding of etiology. Clin Orthop Relat Res.

[3] Klippel M, Feil A (1912). Un cas d’absence des vertèbres cervicales avec cage thoracique remontant jusqu’à la base du crâne. Nouvelle Iconographie de la Salpêtrière.

[4] Clarke RA, Catalan G, Diwan AD, Kearsley JH (1998). Heterogeneity in Klippel–Feil syndrome: a new classification. Pediat Radiol.

[5] McGaughran JM, Oates A, Donnai D, Read AP, Tassabehji M (2003). Mutations in PAX1 may be associated with Klippel–Feil syndrome. European J Human Genet.

[6] Nguyen VD, Tyrrel R (1993). Klippel-Feil syndrome: patterns of bony fusion and wasp-waist sign. Skeletal Radiol.

[7] Illig KA, Donahue D, Duncan A, Freischlag J, Gelabert H, Johansen K (2016). Reporting standards of the Society for Vascular Surgery for thoracic outlet syndrome. J Vasc Surg.

[8] Povlsen B, Hansson T, Povlsen SD (2014). Treatment for thoracic outlet syndrome. Cochrane Database Syst Rev.

[9] Klippel M., Feil A. (1912). Un cas d’absence des vertébres cervicales cage thoracique remontant jusqu’a La Base Du Crâne. Bull Mémoires Soc Anthropol Paris.

[10] Samartzis DD, Herman J, Lubicky JP, Shen FH (2006). Classification of congenitally fused cervical patterns in Klippel-Feil patients: epidemiology and role in the development of cervical spine-related symptoms. Spine (Phila Pa 1976).

[11] Frikha R. (2020). Klippel–Feil syndrome: a review of the literature. Clin Dysmorphol.

[12] Litrenta J., Bi A.S., Dryer J.W. (2021). Klippel–Feil syndrome: pathogenesis, diagnosis, and management. J Am Acad Orthop Surg.

[13] Jae-Min Park A., Nelson S.E., Mesfin A. (2022). Klippel–Feil syndrome: clinical presentation and management. JBJS Rev.

[14] Konstantinou DT, Chroni E, Constantoyiannis C, Dougenis D (2004). Klippel-Feil syndrome presenting with bilateral thoracic outlet syndrome. Spine (Phila Pa 1976).

[15] Hazra D, Sen I, Selvaraj D, Premkumar P, Agarwal S (2018). Arterial thoracic outlet syndrome in Klippel-Feil syndrome. ANZ J Surg.

